# Value-based evaluation of gestational diabetes mellitus care pathway redesign by using cost and outcome data

**DOI:** 10.1186/s12884-025-07576-2

**Published:** 2025-05-26

**Authors:** Maud van den Berg, Julia Spaan, Jacoba van der Kooy, Monique Klerkx, Charlotte Krol, Arie Franx, Kees T.B. Ahaus, Hilco J. van Elten

**Affiliations:** 1 Erasmus School of Health Policy & Management, Erasmus University Rotterdam, Burgemeester Oudlaan 50, Rotterdam, 3062 PA The Netherlands; 2 Obstetrics and Gynaecology, Amphia Hospital, Molengracht 21, Breda, 4818 CK The Netherlands; 3 Department of Obstetrics and Gynaecology, Erasmus Medical Center, Dr. Molewaterplein 40, Rotterdam, 3015 GD The Netherlands; 4 Midwifery Practice, Verloskundigen Oosterhout, Sint Antoniusstraat 86a, Oosterhout, 4902 PV The Netherlands; 5 Internal Medicine, Amphia Hospital, Molengracht 21, Breda, 4818 CK The Netherlands; 6 Department of Accounting, Vrije Universiteit Amsterdam, De Boelelaan 1105, Amsterdam, 1081 HV The Netherlands

**Keywords:** Value-based healthcare, Gestational diabetes mellitus, Service delivery redesign, Time-driven activity-based costing, Clinical outcomes, Patient-reported experience measures

## Abstract

**Background:**

Gestational diabetes mellitus (GDM) is a common complication of pregnancy. Implementation of Value-Based Healthcare (VBHC) to GDM care is worthwhile as traditional GDM care is fragmented and fails to meet the needs of women with GDM. Value of care can be improved through optimization and redesign of the care pathway and implementation of an outcome-based payment model. This study was conducted to perform a value-based evaluation of GDM care pathway redesign by using cost- and outcome data.

**Methods:**

This study was designed as a single center, prospective, observational cohort study. In January 2022, GDM care was redesigned by substituting GDM care activities from an Internal Medicine Department (IMD) to an Integrated Maternity Care Organization (IMCO) in the Netherlands. Women diagnosed with GDM in 2021 were assigned to a pre-intervention cohort (*N* = 264) and those diagnosed in 2022 to a post-intervention cohort (*N* = 407). The impact of the intervention on value of care for women with GDM was evaluated by comparing clinical outcomes, patient-reported experience measures (GDM Responsiveness questionnaire), and costs (Time-Driven Activity-Based Costing) between the cohorts.

**Results:**

Referrals to the IMD for GDM decreased by 84.8% (pre-intervention: 100%, post-intervention: 15.2%, *p* <.001), patient-reported experiences significantly improved (Mean responsiveness pre-intervention: 3.46, post-intervention: 3.63, p: 0.00). Initiation of insulin treatment decreased by 46.8% (pre-intervention: 25.0%, post-intervention: 13.3%, *p* <.001). Maternal- and neonatal clinical outcomes were not different after redesign. Weighted average costs per GDM treatment were 9.7% lower post-intervention (pre-intervention: €168,37, post-intervention: €151,97).

**Conclusions:**

The redesign of GDM care positively impacted value through decreased referrals and improved patient-reported experiences while clinical outcomes remained constant. By de-fragmenting GDM care, cost savings were realized. This study contributes to the improvement of care delivery, particularly in pregnancy and childbirth, by promoting the adoption of comprehensive, value-based evaluations of redesign initiatives and supports the further uptake of VBHC in maternity care.

**Supplementary Information:**

The online version contains supplementary material available at 10.1186/s12884-025-07576-2.

## Background

Gestational diabetes mellitus (GDM) is a common complication of pregnancy defined as ‘diabetes diagnosed in the second or third trimester of pregnancy that was not clearly overt diabetes prior to gestation’ [1]. The occurrence of GDM is associated with short- and long-term adverse outcomes for both mother and neonate [2]. Global prevalence is 14% but varies between continents due to diverging diagnostic criteria and heterogeneous population characteristics [3, 4]. Prevalence is increasing, which makes GDM a global public health issue [5–7]. An unhealthy lifestyle, in terms of inadequate nutrition and exercise, is associated with the development of GDM [8, 9]. Primary strategies for controlling GDM are therefore aimed at nutrition and exercise [4, 8, 10]. When these fail to be effective, pharmacological treatment with insulin is needed [11, 12]. This is required for 15–30% of the women with GDM [13].

Studies have shown that the traditional GDM care pathway is fragmented and fails to meet the needs of women with GDM, highlighting the potential for improving value of care for women with GDM [14–16]. Value-Based Healthcare (VBHC) is concerned with maximizing value for patients. Value is defined as ‘the health outcomes achieved per dollar spent’ and is improved by enhancing outcomes at comparable or lower cost or achieving comparable outcomes at lower cost [17, 18]. Healthcare providers can enhance value by implementing interventions aimed at redesigning care pathways, such as substituting care from a secondary care setting to a primary care setting [19–22]. Additionally, outcome-based payment models can be designed to reward healthcare providers for improving value and further incentivize the practice of VBHC [23–25]. Implementation of VBHC depends on insight into resource utilization and quality of care using cost- and outcome data [19, 26]. However, practical experiences with comprehensively collecting, analyzing, and evaluating these data are scarce [27].

This study was conducted to perform a value-based evaluation of GDM care pathway redesign by using cost- and outcome data. Costs were calculated with Time-Driven Activity-Based Costing and outcomes were collected using clinical outcomes and patient-reported experience measures. This study was part of a 2-year action research project conducted at an Integrated Maternity Care Organization (IMCO) in the Netherlands. The research project was aimed at designing outcome-based payment models for redesigned maternity care delivery pathways based on cost- and outcome data. This study contributes to the improvement of care delivery, particularly in pregnancy and childbirth, by promoting the adoption of comprehensive, value-based evaluations of redesign initiatives and supports the further uptake of VBHC in maternity care.

## Methods

### Study aim

This study’s aim was twofold. First, state-of-the-art methods were applied to collect cost- and outcome data of GDM care. Second, the collected cost- and outcome data were comprehensively evaluated to determine the impact of redesigning the GDM care pathway on value for women with GDM.

### Study design

To compare the value of care before- and after the intervention, the study was designed as a single center, prospective, observational cohort study. The intervention was implemented 1st of January 2022. Two cohorts were created based on a dataset containing clinical outcome data of women who gave birth at the IMCO between 2021 and 2022. The pre-intervention cohort includes women diagnosed with GDM between June 2021 and December 2021, and who gave birth in either 2021 or 2022. The post-intervention cohort includes women diagnosed with GDM between January 2022 (date of implementation) and December 2022, and who gave birth in 2022. Women who were diagnosed with GDM in 2022 and gave birth in 2023 were not included. Both cohorts were compared using clinical outcomes and patient-reported experiences. Clinical outcomes were derived from electronic medical records. Patient experiences were measured with a GDM Responsiveness Questionnaire.

### Study setting

The Dutch maternity care system works as a two-tier model. For low-risk pregnancies, community midwives of independent practices in the neighborhood are in the lead. In this midwifery-led setting, women are allowed to choose to give birth at home or at a midwifery-led outpatient setting in a hospital. Referrals to the obstetrician in the hospital are possible for medical reasons during the antenatal, intrapartum, and post-partum phase. Women with medium to high-risk pregnancies receive obstetric care in an obstetrician-led setting within the hospital [28].

At the IMCO (Annature Geboortezorg in Breda, the Netherlands) women are still allocated to a midwifery-led or obstetrician-led setting based on their estimated risk, but the traditional two tiers are organizationally integrated. It is organized as a network with partners: 13 community midwife practices, 6 maternity nursing organizations, an OB/GYN (Obstetrics & Gynecology) medical specialist enterprise, and the obstetric department of a top clinical teaching hospital (Amphia Hospital in Breda, the Netherlands).

The IMCO is paid via bundled payments, meaning that one single payment encompasses all the services required instead of separate payments for each provider and service [29]. While a bundled payment structure is already considered an innovative provider payment model, the overall research project aimed to develop a shared-savings model explicitly linked to cost and outcome data. In this approach, the IMCO can be rewarded for value-improving efforts and is motivated to continuously optimize care pathways.

### Redesign of GDM care

In the Netherlands, GDM is diagnosed by the obstetric care professional (midwife or obstetrician) using a 75 g oral glucose intolerance test (OGTT). Usually, this is at 16 weeks in case of GDM in a previous pregnancy or between 24 and 28 weeks when risk factors are present, such as body mass index > 30 kg/m2, previous history of GDM, previous infant born > 95th centile, family history of diabetes, high-risk ethnic group, polycystic ovary syndrome, polyhydramnios or suspected macrosomia. GDM is diagnosed if fasting glucose is ≥ 5.1 mmol/L, 1-hour glucose ≥ 10.0 mmol/L, or 2-hour glucose ≥ 7.8 mmol/L. Primary strategies for controlling GDM are aimed at lifestyle management through nutrition therapy and exercise. Nutrition therapy entails a personal diet to regulate carbohydrate intake, designed by a dietician. Frequent monitoring is done to check whether this adequately controls blood sugars. In case of persistent hyperglycemia (fasting glucose ≥ 5.3 mmol/L or postprandial glucose ≥ 7.0 mmol/L in at least two separate measurements) despite adherence to lifestyle changes, treatment with insulin is necessary [30, 31]. Insulin therapy is required for 15–30% of the women with GDM [13]. In the Netherlands, metformin and other oral hypoglycemic agents are not routinely used for the treatment of GDM [32].

Before the redesign intervention, women diagnosed with GDM were referred to the IMD of the hospital to receive GDM care from the internal medicine specialist and diabetes nurse. This was perceived as a burden, as it resulted in two separate care pathways: a perinatal care pathway at the IMCO and a GDM care pathway at the IMD. To overcome this, the obstetric care professionals of the IMCO were trained to deliver GDM care and became responsible for blood sugar monitoring. The IMD is involved only when therapy with insulin is needed, as this requires specific expertise. The criteria for initiating insulin therapy remained consistent with established standards. The intervention improved continuity of care for women with diet and exercise-controlled GDM, as referrals to the IMD are no longer necessary. It was expected that this intervention would lead to a 50% reduction of referrals to the IMD while quality of care and patient experiences remained constant or would improve.

## Outcome measurement

### Study population

#### Primary outcome

The primary outcome was the number of referrals to the IMD, whether related to initiation of insulin therapy or not.

#### Secondary clinical outcomes

Clinical outcomes are categorized into: (1) delivery related outcomes: birth location at start- and end of birth, delivery type, induction of labor, (2) maternal outcomes: excessive maternal weight gained [33], polyhydramnios (amniotic fluid index greater than 24 cm), preeclampsia, gestational hypertension, hemorrhage (blood loss > 1000 ml) and, (3) neonatal outcomes: neonatal weight at delivery (grams), gestational age at delivery (weeks), macrosomia (birth weight equal to or greater than the 90th percentile), preterm birth, Apgar score of less than 7 at 5 min after birth, small-for-gestational-age (birth weight less than the 10th percentile), shoulder dystocia, perinatal death and involvement of pediatrics after birth. Hoftiezer’s centiles for birth weight were used, which are based on actual birth weight distributions from a large Dutch perinatal dataset. The centiles were adjusted for sex and gestational age at birth [34].

#### Secondary patient-reported experiences

The GDM Responsiveness Questionnaire was developed to measure patient experiences with GDM care. The questionnaire was based on the validated Responsiveness in Perinatal and Obstetric Health Care Questionnaire (ReproQ). Responsiveness focusses on the non-clinical part of quality of care and patient experience [35]. The ReproQ was developed to measure responsiveness specifically for perinatal and obstetric care based on the domains: Dignity, Autonomy, Confidentiality, Communication, Prompt attention, Social considerations, Basic amenities and Choice and continuity.

To specifically measure experiences with GDM care, a specification of the ReproQ was carried out by a multidisciplinary team of clinical experts and an epidemiologist. Several questions were added to fit the nature of GDM care. The response rate was 38% in the pre-intervention cohort and 31% in the post-intervention cohort. Questionnaire construct validity was assessed by Harman’s single factor test and reliability tests (% variance explained, Cronbach’s alpha). For all domains, the explained variance was > 0.50%, which was considered acceptable. Cronbach’s alphas ranged between 0.53 and 0.84 and everything above 0.70 was considered acceptable [36]. Two domains resulted below this metric. Cronbach’s alpha has a lower result when the number of items is limited [37]. In that case, interitem correlations can be calculated, which remained within the acceptable range of 0.20-0.40 [38].

#### Analysis

Clinical outcomes and patient-reported experiences were analyzed in SPSS version 29 (IBM Corporation Inc. Armonk, NY, USA). Descriptive statistics were performed to compare sociodemographic information per cohort. For dichotomous variables, univariate Chi-square tests were performed. For continuous variables, independent-samples T-test or Mann-Whitney U-tests were applied, depending on normal distribution. The GDM Responsiveness data were analyzed on the following levels: overall mean questionnaire score, mean score per domain and mean score per item. Independent samples T-tests were applied when Skewness and Kurtosis remained within the acceptable range of +/- 3 and respectively +/- 10 [39]. Means, standard deviations, mean difference and Cohen’s D are reported. Thresholds for Cohen’s D are 0.2 for small, 0.5 medium and 0.8 large effect [40]. P-values below 0.05 were considered significant, values below 0.1 were perceived moderately significant. Nonparametric tests were conducted to ensure robustness, and these tests yielded comparable results.

### Cost measurement

Time-Driven Activity-Based Costing (TDABC) is a cost-accounting method to calculate the costs of care pathways [41]. The method departs from the patient journey and allocates supplied costs to care pathways with time as allocation key [42]. This is done through the calculation of ‘capacity cost rates’ (CCR); the ratio of costs to practical capacity (time available to provide patient care) [43]. TDABC fits the variable nature of healthcare as the costs of most care activities are primarily determined by the amount of time that specific resources are utilized for [44]. Through this bottom-up approach, TDABC aims to be accurate and pragmatic [45]. The method is applied by following a seven-step method, which is detailed below [26, 41].

**Step 1** is the identification of the study’s aim and the medical condition to be costed. It was the aim to compare the costs of the GDM care pathway before and after the intervention. To determine this accurately, a three-level differentiation was applied. Firstly, we differentiated between pre- and post-intervention. Secondly, we differentiated between responsible maternity care setting (midwifery-led or obstetrician-led) at the time of GDM diagnosis. Note that women allocated to the midwifery-led setting can be referred to the obstetrician-led setting at different times during pregnancy due to different kinds of medical indications. To not further complexify the cost calculation, these referrals were not incorporated in the cost calculation. This means that women allocated to the midwifery-led group remained in that group even though they were referred later in pregnancy. Thirdly, we differentiated between GDM treatment with diet and exercise therapy or insulin therapy, as these care processes differ. This led to eight (2*2*2) care pathways to be costed.

Direct observations and interviews before- and after the intervention were conducted to guide step 2 and 3. During **Step 2**, the process of the GDM care pathways before- and after the intervention were mapped at the activity level. Due to the specific focus on costs of GDM care, all non-GDM related obstetric care activities in the overall maternity care pathway were left out. During **Step 3** the supplied resources in the care pathways before- and after the intervention were identified. These were divided between direct (frontline personnel) and indirect resources (building and general unit/practice related overhead costs and non-clinical, administrative and support staff personnel costs).

**Step 4** is the estimation of the total cost of each resource, which was done by extracting cost data from the hospital’s and IMCO’s financial database, the hospitals’ HR system and the database of the Dutch Healthcare Authority (NZa). Hospital-related overhead costs were incorporated using the hospital’s internal cost pricing for GDM care, as indirect cost data at the departmental level were unavailable. **Step 5** is the calculation of the CCRs. CCRs were calculated to allocate frontline personnel costs and overhead costs. For the frontline personnel costs, CCRs were calculated on actor-level (per profession) based on total cost per profession per year divided by their practical capacity per year in minutes. For the overhead costs, a hospital-related overhead costs CCR (similar for IMD and IMCO’s obstetrician-led setting) and a midwifery-led setting-related overhead costs CCR were calculated.

**Step 6** is the collection of time estimates for all care activities in the GDM care pathways, which were obtained with direct observations and interviews. The estimates were validated by expert opinions. Time data collection was carried out before and after intervention. **Step 7** is the calculation of the costs of the GDM care pathways by multiplying the CCRs with the corresponding time estimates of the activities in the care pathways and allocating the direct costs.

For comparison, the Weighted Average Costs (WAC) of GDM treatment pre- and post-intervention were calculated. This is a cost estimate in which the costs of the differentiated care pathways are combined and weighted based on their proportion in the cohort. Percentual change in costs was determined by comparing the WAC of GDM treatment before and after the intervention.

### Value-assessment

**Fig. 1:**
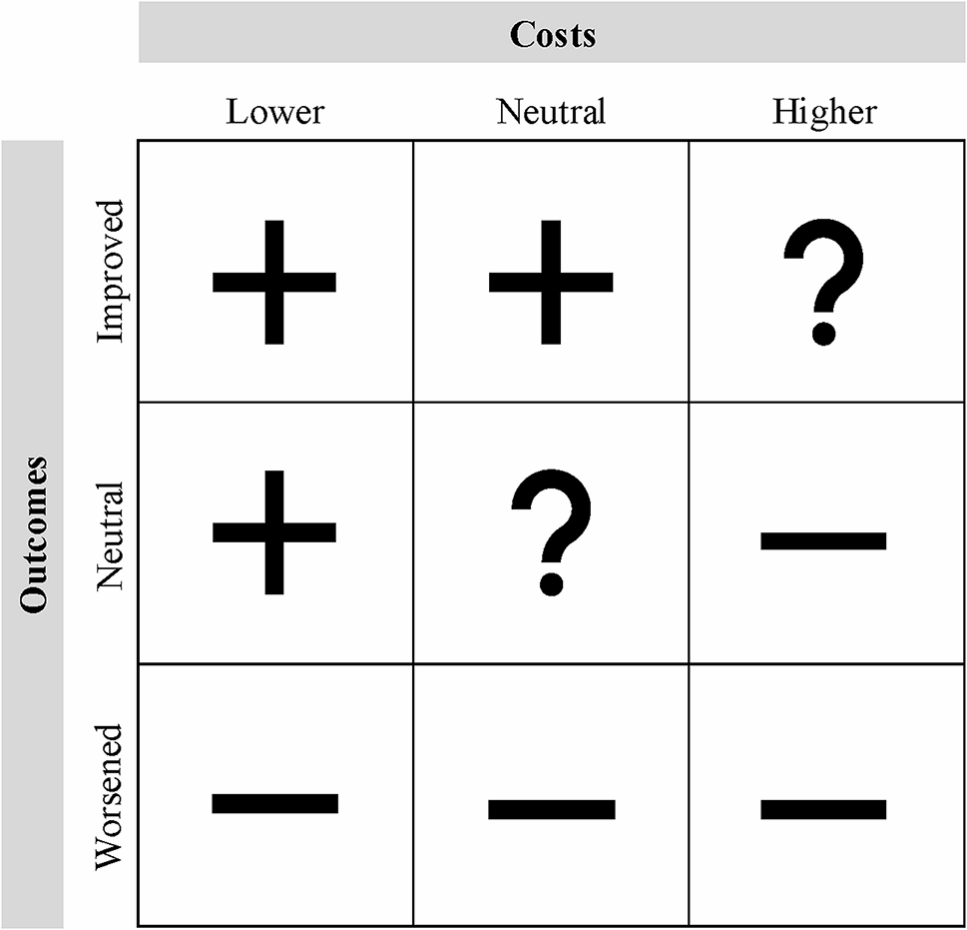
Value-assessment matrix and corresponding evaluations per scenario

A value-assessment matrix (Fig. 1) was developed to visually combine the impact on costs- and outcomes and perform the value-based evaluation of the implemented GDM care pathway redesign. The columns represent the change in costs calculated with TDABC and the rows represent the change in outcomes, which is a combination of the clinical outcomes and patient-reported experiences. Three types of evaluations were possible, namely a positive impact on value (+), a negative impact on value (-) and an unconvincing impact on value (?). In this study, the *neutral outcome change / neutral cost change* and the *improved outcome change / higher costs* scenarios were considered unconvincing. In these cases, the intervention did not have a negative nor positive impact. A repeated evaluation after a specific time is then suggested.

## Results

Sociodemographic characteristics of the pre- and post-intervention cohorts are presented in Table 1, which shows that there are no significant differences between the two groups.

**Table 1:** Patient characteristics pre- and post-intervention cohorts

	Pre-intervention	Post-intervention	*p*
Participants, *n*	264	407	
Age, *mean* ± *SD*	32 ± 4,5	32 ± 4,8	0.97
Para, *n* (%)			
Nulli	107 (40.5)	179 (44.0)	0.38
Ethnic background, *n* (%)			
Other than Caucasian	66 (25.0)	102 (25.1)	0.98
Proficiency (speaking) Dutch, *n* (%)			
Not sufficient	22 (8.3)	45 (11.1)	0.25
Neighborhood, *n* (%)			
Underprivileged	24 (9.1)	42 (10.3)	0.60
Body mass index, *n* (%)			
< 25	83 (31.4)	132 (32.5)	0.85
25–29,99	83 (31.4)	132 (32.5)	
> 30	98 (37.2)	142 (35.0)	

### Primary outcome

After the intervention, the number of referrals to the IMD decreased significantly from 100% to 15.2%. Furthermore, the number of women treated with insulin decreased significantly from 25% to 13.3% (Table 2).

**Table 2:** Primary and secondary clinical outcomes pre- and post-intervention cohorts

	Pre-intervention (*N* = 264)	Post-intervention (*N* = 407)	*p*
**Primary outcomes**			
Referral to internal medicine, *n* (%)	264 (100.0)	62 (15.2)	< 0.001
Insulin therapy for GDM, *n* (%)	66 (25.0)	54 (13.3)	< 0.001
**Secondary outcomes**			
***Delivery related outcomes***			
Birth location: start of birth, *n* (%)			0.55
IMCO: midwifery-led	76 (28.8)	125 (30.9)	
IMCO: obstetrician-led	188 (71.2)	279 (69.1)	
Birth location: end of birth, *n* (%)			0.88
IMCO: midwifery-led	40 (15.2)	60 (14.7)	
IMCO: obstetrician-led	224 (84.8)	352 (85.3)	
Delivery type, *n* (%)			0.71
Spontaneous vaginal	190 (72.0)	281 (69.0)	
Assisted vaginal	17 (6.4)	30 (7.4)	
Caesarean section	57 (21.6)	96 (23.6)	
Caesarean section, *n* (%)			0.79
Planned	27 (10.2)	43 (10.6)	
Unplanned	30 (11.4)	53 (13.1)	
Induced labor, *n* (%)	125 (47.5)	190 (46.7)	0.83
***Maternal outcomes***			
Excessive Gestational weight gain, *n* (%)	86 (32.6)	117 (28.9)	0.30
Polyhydramnios, *n* (%)	13 (4.9)	16 (3.9)	0.54
Preeclampsia, *n* (%)	1 (0.40)	3 (0.7)	-
Gestational hypertension, *n* (%)	12 (4.5)	17 (4.2)	0.82
Hemorrhage (> 1000 ml), *n* (%)	22 (8.3)	23 (5.7)	0.18
***Neonatal outcomes***			
Neonatal weight, g *(mean ± SD)*	3459 ± 512	3466 ± 526	0.54
GA, weeks *(mean ± SD)*	38.8 ± 1.3	38.7 ± 1.6	0.74
Macrosomia (> p90), *n* (%)	40 (15.2)	58 (14.3)	0.75
Preterm birth (< 37 weeks), *n* (%)	12 (4.5)	26 (6.4)	0.31
5-minute APGAR (< 7), *n* (%)	8 (3.0)	11 (2.7)	0.81
SGA (< p10), *n* (%)	23 (8.7)	35 (8.6)	0.96
Shoulder dystocia, *n* (%)	2 (0.8)	4 (1.0)	-
Perinatal death, *n* (%)	0 (0.0)	2 (0.5)	-
Pediatrics involvement, *n* (%)	193 (73.1)	269 (67.1)	0.10

IMCO (Integrated Maternity Care Organization), GA (Gestational Age), APGAR (Appearance, Pulse, Grimace, Activity and Respiration), SGA (Small for Gestational Age)

### Secondary clinical outcomes

The redesign of the GDM care pathway had no significant impact on birth-related and clinical outcomes for mother and neonate (Table 2). Regarding the perinatal death cases, one stillbirth was associated with severe preeclampsia (25 weeks of gestation), and the other stillbirth resulted from placental failure due to extensive perivillous fibrin deposition (34 weeks of gestation). These outcomes are unlikely to be linked to the redesign of the GDM care pathway, as these complications are more directly related to underlying (placental) pathology than altered GDM care delivery.

### Secondary patient-reported experiences

**Fig. 2:**
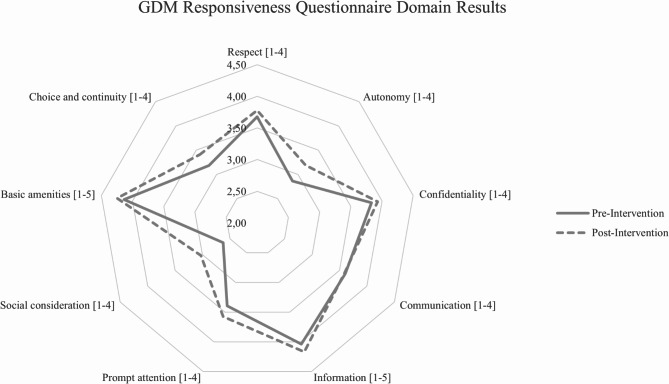
GDM Responsiveness Questionnaire Domain results

The analysis revealed a significant improvement (*p* =.00) in overall responsiveness for the women in the new GDM care pathway (µ = 3.63, *n* = 137) compared to women in the old GDM care pathway (µ = 3.46, *n* = 100) (Table 3; Fig. 2). The intervention had the largest, significant, positive impact on the domain Social consideration as compared to the other domains. Other significant positively impacted domains are Autonomy, Confidentiality, Choice and continuity and Prompt attention. However, the effect sizes of these differences were small to medium. A moderately significant improvement was found for the domains Respect, Information, and Basic Amenities. No significant difference was found for the domain Communication. Scores and test results per item are provided in the appendix (Additional file 1). Patient demographics of responses per cohort were compared between responders pre- and post-intervention, revealing no significant difference. A similar comparison between responders and non-responders indicated significant differences in the determinants of ethnic background and Dutch language proficiency. The results are shared in (Additional file 2).

**Table 3:** GDM responsiveness questionnaire results pre- and post-intervention cohorts

	Pre-Intervention (*N* = 100)	Post-intervention (*N* = 137)	Mean Difference	*P*	Cohen’s D
Domain scores (mean, ±SD)					
Respect [1–4]	3,68 ± 0,45	3,78 ± 0,38	0,10	0.07*	0,2
Autonomy [1–4]	2,86 ± 0,87	3,19 ± 0,77	0,32	0.00**	0,4
Confidentiality [1–4]	3,83 ± 0,37	3,93 ± 0,22	0,10	0.02**	0,3
Communication [1–4]	3,60 ± 0,50	3,59 ± 0,47	-0,02	0.80	0,0
Information [1–5]	4,04 ± 0,58	4,17 ± 0,61	0,13	0.10*	0,2
Prompt attention [1–4]	3,39 ± 0,58	3,58 ± 0,47	0,18	0.01**	0,3
Social consideration [1–4]	2,62 ± 0,61	3,03 ± 0,71	0,41	0.00**	0,6
Basic amenities [1–5]	4,14 ± 0,55	4,25 ± 0,59	0,11	0.14*	0,2
Choice and continuity [1–4]	3,19 ± 0,73	3,41 ± 0,65	0,23	0.01**	0,3
Overall score (mean, ±SD) [1–4,2]	3,46 ± 0,45	3,63 ± 0,40	0,17	0.00**	0,4

P-values < 0.05 were considered significant (**), P-values < 0.1 were perceived moderately significant (*)

### Costs of GDM care

TDABC was applied to calculate the costs of providing GDM care per patient pre- and post-intervention. Based on this analysis, the change in costs of GDM care due to the intervention was determined. Before the intervention, the WAC per GDM care pathway was €168,37 and after the intervention it was €151,97, which is 9.7% lower. Table 4 provides a detailed overview of the calculated costs of the eight separate care pathways and the calculation of the WAC pre- and post-intervention (process maps are provided in Additional file 3).

**Table 4:**
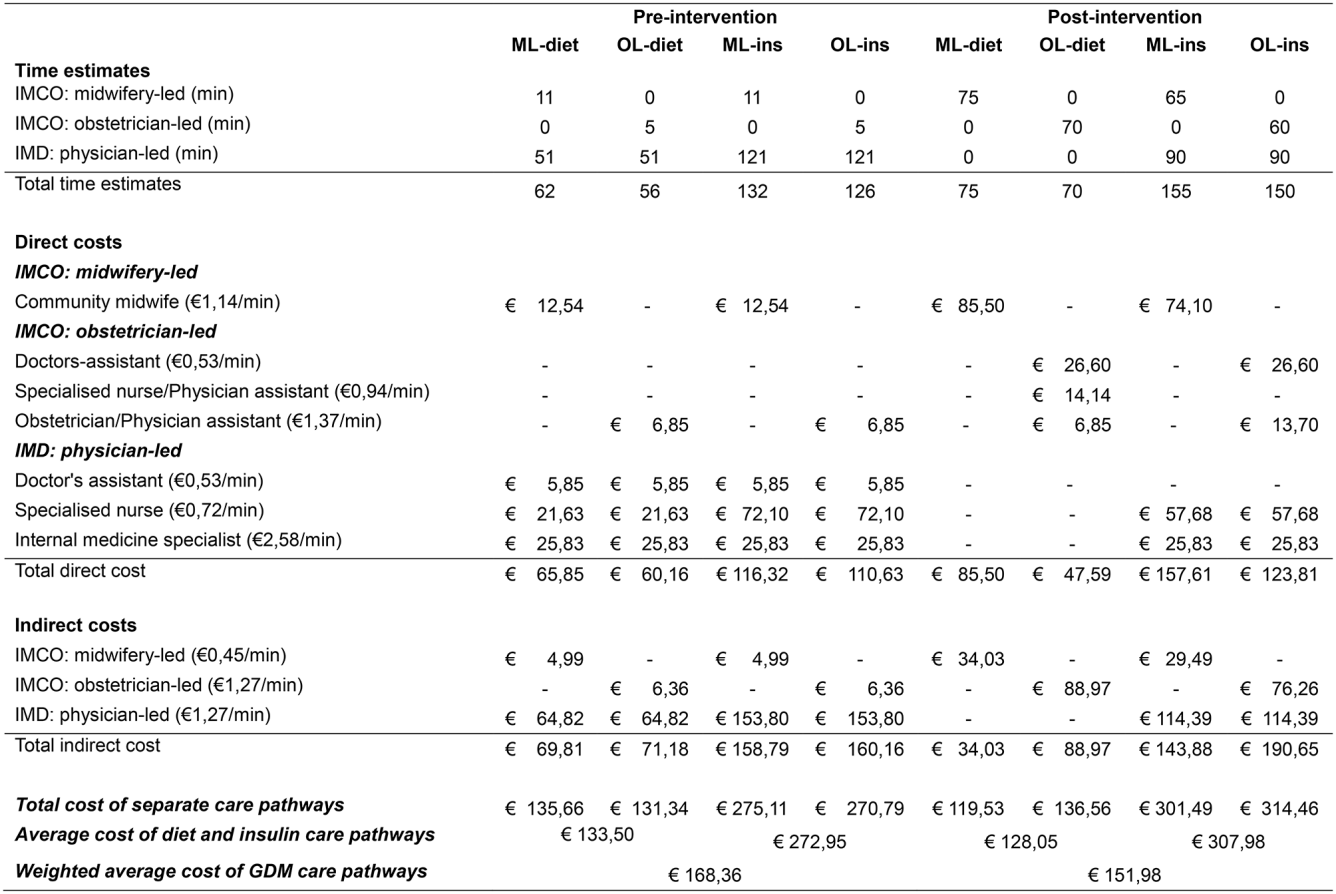
TDABC cost calculation GDM care pre- and post-intervention

Costed care pathways at moment of diagnosis of GDM: ML-diet (midwifery-led, diet), OL-diet (obstetrician-led, diet), ML-ins (midwifery-led, insulin), OL-ins (obstetrician-led, insulin). Physician-led care is applicable in all pre-intervention pathways and only in the case of ML-ins and OL-ins post-intervention. IMCO (Integrated Maternity Care Organization), IMD (Internal Medicine Department)

## Discussion

This study was conducted to perform a value-based evaluation of GDM care pathway redesign by using cost- and outcome data. The intervention of transferring the majority of GDM care to the IMCO resulted in a significant 84.8% reduction in referrals to the IMD and a significant improvement of patient-reported experiences, while clinical outcomes remained stable. Moreover, a cost savings of 9.7% was realized. These findings indicate that the intervention had a positive impact on value of care for women with GDM.

In current literature, comprehensive, value-based evaluations of redesign initiatives in GDM care are scarce. Syrop et al., (2021) redesigned care delivery for insulin-requiring women with GDM by incorporating remote glucose monitoring. Their study showed improved maternal and neonatal clinical outcomes, lower length of stay and costs [20]. In Australia, a digital model of care for GDM has also been implemented, showing similar outcomes to standard, traditional care [46]. Furthermore, it had a positive cost implication for the population [47]. Notably, patient-reported experiences were not an integral part in these evaluations. Redesign initiatives can be successful in terms of cost savings or improved clinical outcomes, but when patient experiences unknowingly deteriorate, the impact on patient value is questionable.

It is key to incorporate outcomes that matter to patients in value-based evaluations. Studies have shown that women with GDM desire a holistic approach in the care process, characterized by emotional, social, and economic components [14, 16]. In the new model of GDM care in this study, these needs were better met. The results of the GDM Responsiveness Questionnaire indicate that women with GDM appreciate the new care pathway. Since the intervention, women feel more empowered in terms of autonomy and choice and appreciate the perceived accessibility of the IMCO’s obstetric care professionals. These professionals provide GDM care in a way that moves beyond the traditional medical approach in terms of attention for the women’s personal situation and social support system. That is because obstetric care professionals are in the unique position to build a partnership with women during pregnancy and thereby are able to better address these needs [48, 49].

The significant decrease in insulin therapies was an unexpected outcome. If this reduction were due to a decline in quality of GDM care provided by the obstetric care professional during pregnancy – for example by inadequate blood sugar monitoring – an increase in GDM-related complications might be expected, such as polyhydramnios and macrosomia. Since we did not observe such an increase, we assume that quality of care was not affected and glycemic control during pregnancy remained adequate. However, as our study was not specifically powered to assess non-inferiority of clinical outcomes, these findings should be interpreted with caution. Further research is needed to formally establish non-inferiority. Perhaps the improved continuity of care positively impacted adherence to the diet and exercise therapy which resulted in a decrease of the number of women in whom insulin therapy was indicated [50]. However, this also requires further research.

The application of TDABC helped to unravel the inherent complexities of organizational costs involved with various healthcare professionals contributing to the GDM care pathway. Even though application of TDABC can be time-consuming, it provides invaluable insights. For example, it is often assumed that transferring care activities from a clinical care setting to a primary care setting has the potential to realize cost savings as the primary care setting is typically less expensive [51–53]. We observed that while the average costs associated with diet therapy provided by the IMCO were indeed lower, the costs for insulin therapy were higher due to the combined efforts required from both the IMCO and the IMD. Notably, the overall cost savings were primarily driven by the significant reduction in the number of insulin therapies.

The value-assessment matrix applied in this study is an intuitive and pragmatic tool to map the impact of interventions on value. Furthermore, it necessitates the incorporation of both critical inputs, as the impact on value cannot be determined when cost- or outcome data are omitted. The matrix also supported this research project in creating unity of language. The overall research project was aimed at designing an outcome-based payment model, which required interdisciplinary collaboration between managers, medical professionals, healthcare insurers and policy makers. The matrix supported in creating a shared understanding of the value concept and the evaluation of the redesign initiative.

Our study has several strengths. Firstly, studies describing real-world cases of extensive redesign of care delivery services are scarce. Therefore, this paper contributes to current literature on the impact of service delivery redesign on value, specifically for GDM care. Secondly, studies performing value-based evaluations with the use of clinical outcomes, patient-reported experience measures and costs of care are limited. This study applied state-of-the-art methods such as a validated questionnaire to specifically measure the experiences of women with GDM care and TDABC to calculate an accurate estimate of costs of GDM care. Thirdly, this study developed and applied an intuitive and pragmatic value-assessment matrix to determine the impact of (redesign) interventions on value which supports the uptake of VBHC in practice.

Our findings must be interpreted within the limitations of this study. First, the GDM Responsiveness questionnaire was only available for Dutch speaking women. Analysis of demographic determinants between responders and non-responders showed a significant difference for determinants ethnic background and non-Dutch speaking women. This might result in bias, as the experiences of these women remain unheard. Second, we did not calculate the costs of the full maternity care pathway, which covers the prenatal, intrapartum, and post-partum period. Larger cost savings could have been detected when including also the intrapartum and post-partum period. Future studies could also expand the cost calculation by including the impact on long-term costs. Third, it was the aim of this study to gain insight into the impact of redesigning GDM care on value on the level of the GDM population. This paper illustrates how cost- and outcome data can be evaluated for this end. The impact on societal value was beyond the scope of this study. Future studies could explore different methodologies to address this aspect, such as a cost-effectiveness study.

Our study has several implications. Firstly, as GDM is a serious public health problem due to its impact on short- and long-term outcomes of mother and neonate, it is of paramount importance to strive for a model of care that improves these outcomes [2, 12]. Furthermore, prevalence of GDM is increasing worldwide [5, 6]. Countries are struggling with growing healthcare costs; therefore, it is necessary to allocate resources efficiently. This study shows that redesigning services results in lower costs while outcomes remain stable, offering an efficient solution. Secondly, this study shows the importance of comprehensively evaluating redesign initiatives. Inputs such as outcomes, patient-reported experiences and costs need to be included in this evaluation. From an organizational perspective, the evaluation could be expanded by also including data on workforce pressures and work satisfaction. Due to the intervention, the IMCO’s professionals have more responsibilities, while these professionals’ time is already under pressure. Therefore, it is important to also consider such factors in the value equation. However, this would complicate the assessment of the overall impact. Therefore, future studies could investigate how the value equation can be expanded by including factors beyond costs and outcomes.

## Conclusion

Gestational diabetes mellitus (GDM) care pathway redesign had a positive impact on value of care for women with GDM. The transfer of low-risk GDM care from the outpatient clinic of the Internal Medicine Department (IMD) to an Integrated Maternity Care Organization (IMCO) resulted in an 84.8% reduction in referrals to the IMD, a 46.8% reduction of initiated insulin therapies and significantly improved patient experiences. Quality of care, measured with clinical, birth-related, maternal, and neonatal outcomes, remained stable. By de-fragmenting the care pathway, cost savings of 9.7% were realized. This paper shows how redesign initiatives have the potential to improve value of care and how state-of-the-art methods can be applied to perform a comprehensive, value-based evaluation. This should support the further uptake of Value-Based Healthcare (VBHC) in maternity care.

## Electronic supplementary material

Below is the link to the electronic supplementary material.

Additional file 1 presents the results of the GDM Responsiveness Questionnaire per item per cohort.

Additional file 2 presents demographic information of responders and non-responders of the GDM Responsiveness Questionnaire

Additional file 3 presents the process maps of the care pathways on activity level.

## Data Availability

The data, models, and methodology used in this research are subject to confidentiality and cannot be shared publicly.

## Abbreviations

GDM: Gestational Diabetes Mellitus
VBHC: Value–Based Healthcare
IPU: Integrated Practice Unit
IMCO: Integrated Maternity Care Organization
IMD: Internal Medicine Department
TDABC: Time–Driven Activity–Based Costing
CCR: Capacity Cost Rate
WAC: Weighted Average Cost
