# Supplementary material for: Value-based evaluation of gestational diabetes mellitus care pathway redesign by using cost and outcome data
Source: BMC Pregnancy Childbirth. 2025 May 26;25:608. doi: 10.1186/s12884-025-07576-2 (PMC12105306; doi:10.1186/s12884-025-07576-2)
Supplement: Supplementary file 1 — Additional file 1 presents the results of the GDM Responsiveness Questionnaire per item per cohort. [file 12884_2025_7576_MOESM1_ESM.pdf]

		Pre-Intervention			Post-intervention					
**Domains**	**Items per domain**	**N**	**M**	**SD**	**N**	**M**	**SD**	**p**	**Mean Difference**	**Cohen's D**
Respect	Did your healthcare professionals treat you with respect?	100	3,87	0,37	137	3,92	0,27	.254	0,050	0,2
	Did your healthcare professionals treat you kindly?	100	3,80	0,45	137	3,85	0,40	.400	0,047	0,1
	Did you feel that you could tell your healthcare professionals everything?	100	3,56	0,72	137	3,67	0,56	.196	0,112	0,2
	Did you receive personal attention from your healthcare professionals?	100	3,51	0,69	137	3,74	0,51	.005	0,235	0,4
	Did your healthcare professionals take your wishes and customs regarding pregnancy into account?	100	3,64	0,63	137	3,71	0,58	.392	0,068	0,1
Autonomy	Did you participate in deciding on your treatment for gestational diabetes if possible?	100	2,91	1,10	137	3,16	0,96	.064	0,251	0,2
	Were you sufficiently involved in discussing blood sugar values?	100	3,21	1,04	137	3,52	0,75	.012	0,308	0,3
	Were you able to refuse a proposed treatment for your gestational diabetes?	100	2,47	1,20	137	2,88	1,16	.008	0,413	0,3
Confidentiality	Did your healthcare professionals take your privacy into account?	100	3,83	0,43	137	3,93	0,28	.034	0,104	0,3
	Did your healthcare professionals handle your medical information and records carefully?	100	3,81	0,42	137	3,93	0,25	.009	0,124	0,4
	Could you discuss important matters with your healthcare professionals without others hearing?	100	3,85	0,44	137	3,92	0,27	.160	0,070	0,2
Communication	Did your healthcare professionals answer your questions about gestational diabetes?	100	3,65	0,61	137	3,73	0,49	.282	0,080	0,1
	Did you understand the explanation from your dietitian?	100	3,83	0,43	137	3,75	0,54	.215	-0,078	-0,2
	Did you understand the explanation from your healthcare professional about gestational diabetes?	100	3,69	0,56	137	3,71	0,54	.804	0,018	0,0
	Did you understand the explanation from your healthcare professionals about monitoring blood sugars?	100	3,74	0,56	137	3,72	0,58	.744	-0,025	0,0
	Did your healthcare professionals give the same advice?	100	3,35	0,80	137	3,34	0,78	.891	-0,014	0,0
	Did your healthcare professional have enough time to explain the different treatment options?	100	3,40	0,95	137	3,45	0,82	.696	0,045	0,1
	Did your healthcare professional tell you what to do if your blood sugar was too high or too low?	100	3,57	0,81	137	3,43	0,85	.203	-0,139	-0,2
Information	How did you find the clarity of the written information (information leaflet) about gestational diabetes?	95	4,08	0,66	129	4,15	0,66	.482	0,063	0,1
	How did you find the clarity of the animation about gestational diabetes?	50	3,90	0,65	86	4,12	0,60	.052	0,216	0,3
	How did you find the user-friendliness of the app for tracking your blood sugar values?	23	4,17	0,65	109	4,30	0,73	.433	0,129	0,2
Prompt attention	Did your healthcare professionals have time for you if you asked for it?	100	3,48	0,70	137	3,64	0,55	.057	0,162	0,3
	Could you quickly make an appointment if it was urgent?	100	3,34	0,81	137	3,64	0,63	.002	0,302	0,4
	Could you easily make an appointment if it was not urgent?	100	3,29	0,83	137	3,53	0,62	.015	0,243	0,3
	Were you quickly seen at your appointment?	100	3,26	0,79	137	3,47	0,65	.023	0,214	0,3
	Was your dietitian easily accessible?	96	3,63	0,58	130	3,52	0,74	.249	-0,102	-0,2
	Was your healthcare professional for gestational diabetes easily accessible?	100	3,38	0,79	137	3,63	0,64	.011	0,248	0,3
Social consideration	Did your healthcare professionals take your situation into account, such as your family, work, and daily activities?	100	2,71	0,98	137	3,11	0,98	.002	0,399	0,4
	Did you feel supported by your partner, family, or others?	100	3,61	0,63	137	3,64	0,62	.761	0,025	0,0
	Were your partner or family involved in the care for your gestational diabetes?	100	1,55	0,97	137	2,34	1,26	.000	0,793	0,7
Basic amenities	Were the waiting room and consultation room in the midwifery practice comfortable?	84	4,36	0,63	131	4,43	0,66	.438	0,070	0,1
	Were the waiting room and consultation room in the hospital comfortable?	78	4,01	0,75	110	4,13	0,83	.331	0,114	0,1
	How did you find the availability of glucose strips and glucose meters?	100	4,06	0,85	137	4,14	0,91	.500	0,079	0,1
Choice and continuity	Did your healthcare professionals give the same advice about your gestational diabetes?	100	3,38	0,74	137	3,40	0,76	.828	0,021	0,0
	When there was a change of healthcare professionals, was your new healthcare professional well informed about your situation?	100	3,31	0,90	137	3,43	0,81	.280	0,121	0,1
	Was it always clear who was in charge of the care you received for gestational diabetes?	100	2,67	1,15	137	3,23	1,00	.000	0,556	0,5
	Was your health and that of your baby monitored?	100	3,38	0,94	137	3,58	0,70	.069	0,204	0,2
