# Supplementary material for: Value-based evaluation of gestational diabetes mellitus care pathway redesign by using cost and outcome data
Source: BMC Pregnancy Childbirth. 2025 May 26;25:608. doi: 10.1186/s12884-025-07576-2 (PMC12105306; doi:10.1186/s12884-025-07576-2)
Supplement: Supplementary file 2 — Additional file 2 presents demographic information of responders and non-responders of the GDM Responsiveness Questionnaire [file 12884_2025_7576_MOESM2_ESM.pdf]

	Non-responders	Responders	p
Participants, *n*	504 (100)	166 (100)	
Age, *mean*	32	33	.015
Para, *n* (%)			
Nulli	204 (40.5)	82 (49.4)	.044
Ethnic background, *n* (%)			
Other than Caucasian	153 (30.4)	15 (9.0)	<.001
Proficiency (speaking) Dutch, *n* (%)			
No	61 (12.1)	5 (3.0)	<.001
Neighborhood, *n* (%)			
Underprivileged	55 (10.9)	11 (6.6)	.108
Body mass index, *n* (%)			
<25	168 (33.4)	47 (28.3)	.304
25-29,99	163 (32.4)	52 (31.3)	
>30	172 (34.2)	67 (40.4)	
