# Supplementary material for: Value-based evaluation of gestational diabetes mellitus care pathway redesign by using cost and outcome data
Source: BMC Pregnancy Childbirth. 2025 May 26;25:608. doi: 10.1186/s12884-025-07576-2 (PMC12105306; doi:10.1186/s12884-025-07576-2)

Pre-intervention: Gestational Diabetes Mellitus – diet care pathway

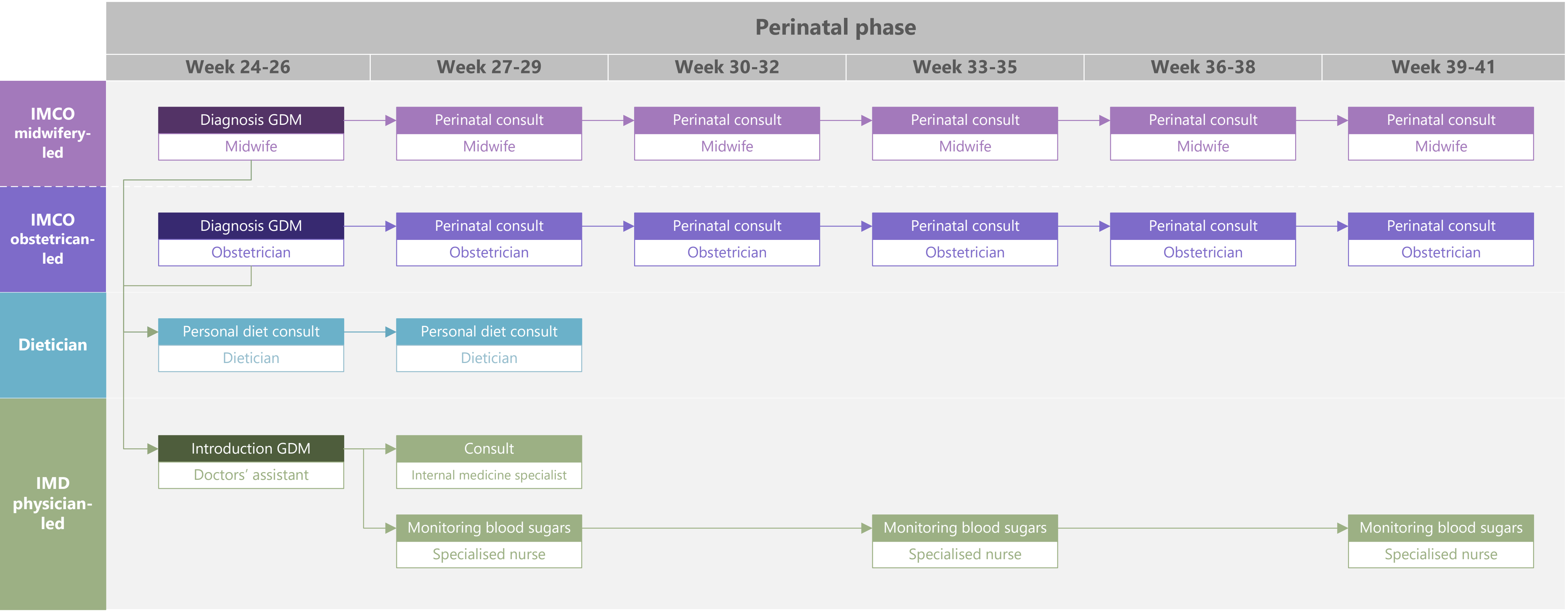

Pre-intervention: Gestational Diabetes Mellitus – insulin care pathway

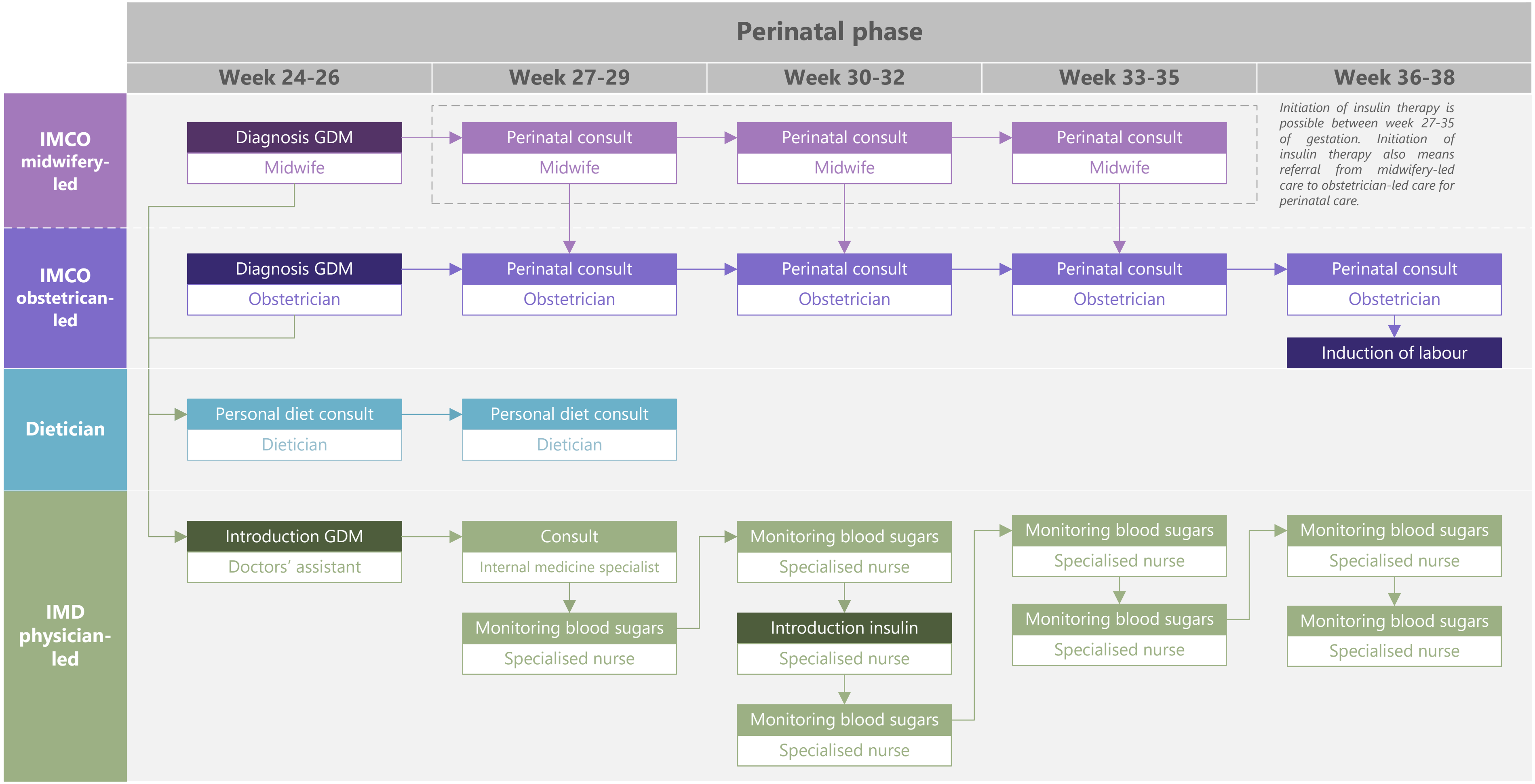

Post-intervention: Gestational Diabetes Mellitus – diet care pathway

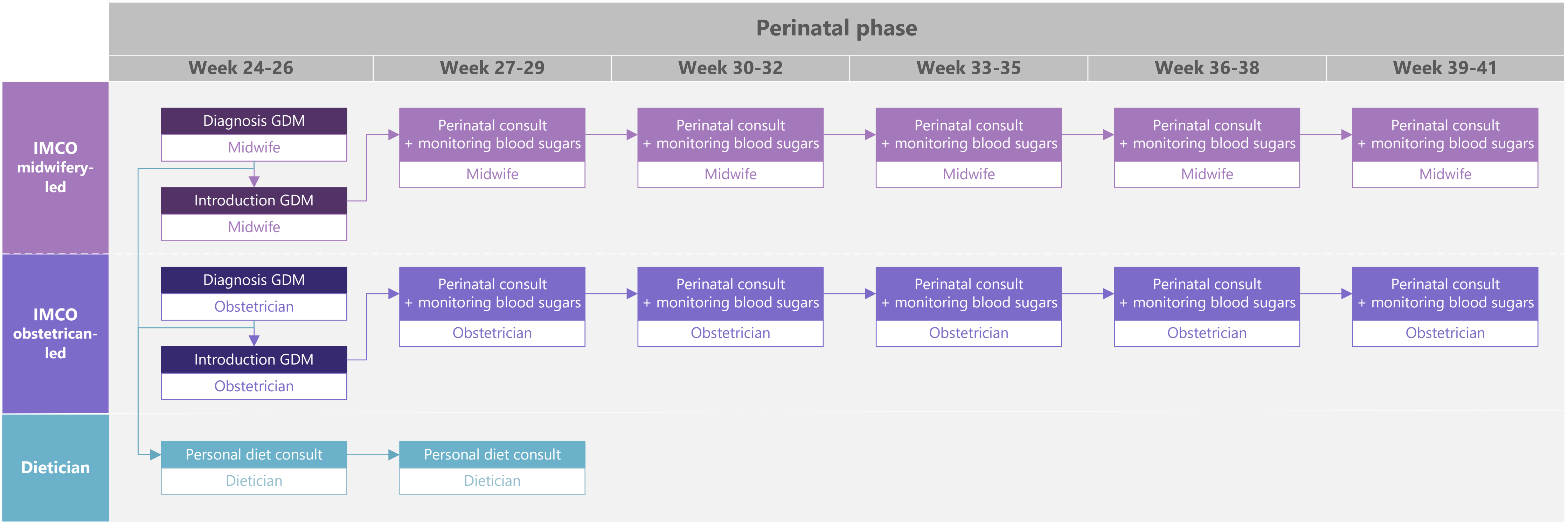

Post-intervention: Gestational Diabetes Mellitus – insulin care pathway

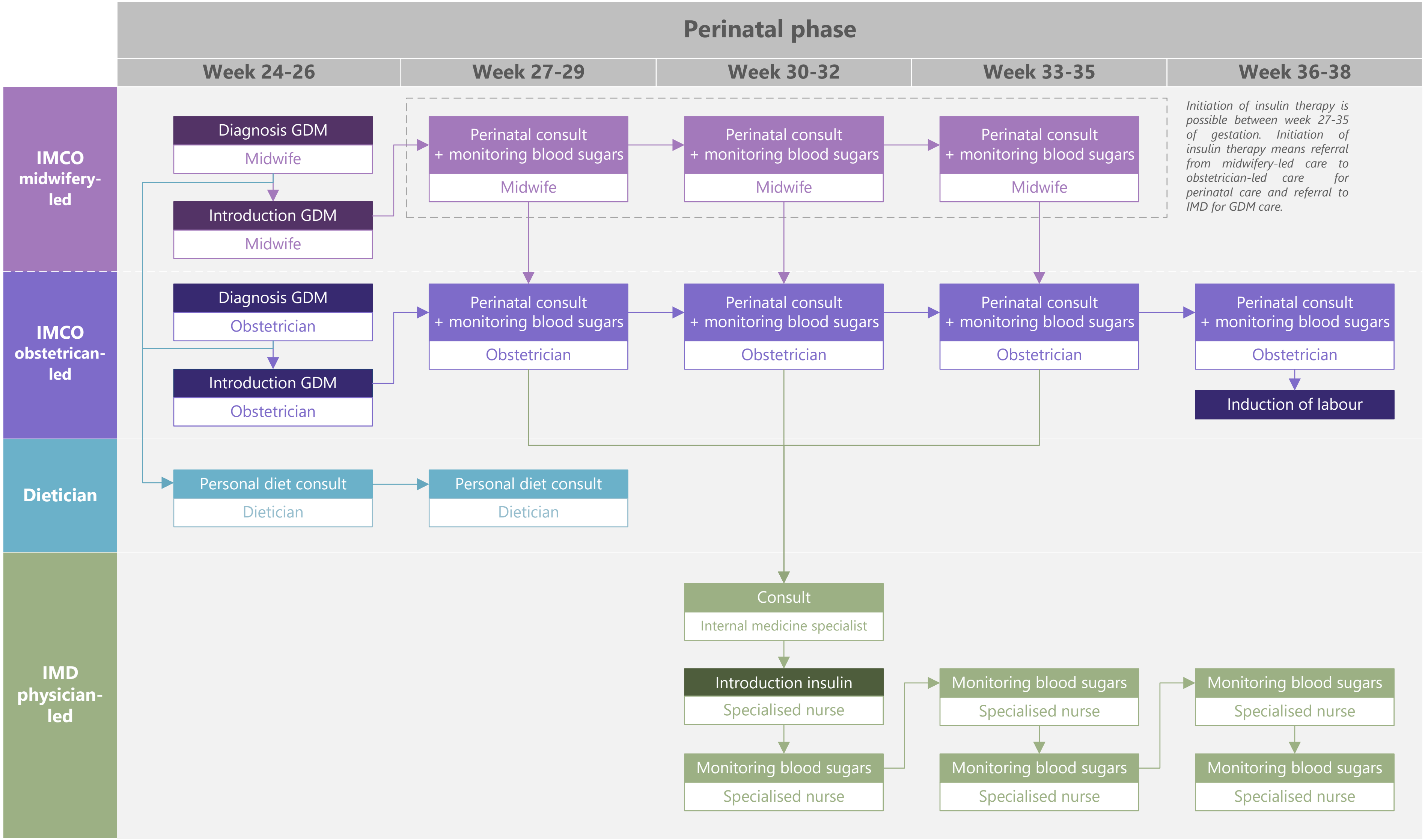

Supplement: Supplementary file 3 — Additional file 3 presents the process maps of the care pathways on activity level. [file 12884_2025_7576_MOESM3_ESM.pdf]
